# Effects of Exercise Training Interventions on Executive Function in Older Adults: A Systematic Review and Meta-Analysis

**DOI:** 10.1007/s40279-020-01292-x

**Published:** 2020-05-23

**Authors:** Feng-Tzu Chen, Jennifer L. Etnier, Kuei-Hui Chan, Ping-Kun Chiu, Tsung-Ming Hung, Yu-Kai Chang

**Affiliations:** 1 Graduate Institute of Sport, Leisure and Hospitality Management, National Taiwan Normal University, Taipei, Taiwan ROC; 2 Department of Kinesiology, University of North Carolina at Greensboro, Greensboro, NC USA; 3 Graduate Institute of Athletics and Coaching Science, National Taiwan Sport University, Taoyuan, Taiwan ROC; 4 Department of Physical Education, National Taiwan Normal University, 162, Section 1, Heping E. Rd., Taipei, Taiwan ROC; 5 Institute for Research Excellence in Learning Science, National Taiwan Normal University, Taipei, Taiwan ROC

## Abstract

**Background:**

Chronic exercise training has been shown be to positively associated with executive function (EF) in older adults. However, whether the exercise training effect on EF is affected by moderators including the specific sub-domain of EF, exercise prescription variables, and sample characteristics remains unknown.

**Objectives:**

This systematic and meta-analytic review of randomized controlled trials (RCTs) investigated the effects of exercise training on EF in older adults and explored potential moderators underlying the effects of exercise training on EF.

**Methods:**

In accordance with the PRISMA guidelines, the electronic databases MEDLINE (PubMed) and EMBASE (Scopus) were searched from January 2003 to November 2019. All studies identified for inclusion were peer-reviewed and published in English. To be included, studies had to report findings from older (> 55 years old), cognitively normal adults or adults with mild cognitive impairment (MCI) randomized to an exercise training or a control group. The risk of bias in each study was appraised using the Cochrane risk-of-bias tool. Fixed-effects models were used to compare the effects of exercise training and control conditions on EF assessed at baseline and post-intervention. In addition, subgroup analyses were performed for three moderators (i.e., the specific sub-domain of EF, exercise prescription variables, and sample characteristics).

**Results:**

Thirty-three RCTs were included. Overall, exercise training was associated with a significant small improvement in EF [*Q*(106) = 260.09, Hedges’ *g* = 0.21; *p* < 0.01]. The EF sub-domain moderator was not significant [*Q*(2) = 4.33, *p* > 0.05], showing that the EF improvement in response to exercise is evident for measures of inhibition, updating, and shifting. Regarding exercise prescription variables, results were significantly moderated by frequency of exercise training [*Q*(1) = 10.86, *p* < 0.05], revealing that effect sizes (ESs) were larger for moderate frequency (*g* = 0.31) as compared to low frequency exercise (*g* = 0.15). The results also showed type of exercise training moderated the ESs [*Q*(4) = 26.18, *p* < 0.05], revealing that ESs were largest for other forms of exercise (*g* = 0.44), followed by Tai Chi and yoga (*g* = 0.38), resistance exercise (*g* = 0.22), aerobic exercise (*g* = 0.14), and combined exercise (*g* = 0.10). In addition, The results showed moderated length of training the ESs [*Q*(2) = 16.64, *p* < 0.05], revealing that ESs were largest for short length (*g* = 0.32), followed by mid length (*g* = 0.26) and long length (*g* = 0.09). No significant difference in effects was observed as a function of exercise intensity [*Q*(1) = 2.87 *p* > 0.05] and session time [*Q*(2) = 0.21, *p* > 0.05]. Regarding sample characteristics, the results were significantly moderated by age [*Q*(2) = 20.64, *p* < 0.05], with significant benefits for young-old (55–65 years old) (*g* = 0.30) and mid-old (66–75 years old) (*g* = 0.25), but no effect on EF for old-old (more than 75 years old). The results were also significantly moderated by physical fitness levels [*Q*(1) = 10.80, *p* < 0.05], revealing that ESs were larger for sedentary participants (*g* = 0.33) as compared to physically fit participants (*g* = 0.16). In addition, results were also significantly moderated by cognitive status [*Q*(1) = 11.44, *p* < 0.05], revealing that ESs were larger for participants with cognitively normal (*g* = 0.26) as compared to those with mild cognitive impairment (*g* = 0.08). No significant differences in effects were observed as a function of sex [*Q*(2) = 5.38, *p* > 0.05].

**Conclusions:**

Exercise training showed a small beneficial effect on EF in older adults and the magnitude of the effect was different across some moderators.

## Key Points

**Table:** 

Exercise training improves multiple domains of executive function (EF), including inhibition, updating, and shifting.
Exercise training leads to improved EF regardless of frequency, intensity, type, session time, length, and sex for all age groups except those over 75 years of age.
Exercise training with a frequency of 3–4 times per week, with vigorous intensity, and other forms of exercise/Tai Chi and yoga, as well as a length of training period from 1 to 3 months, produces larger beneficial effects on EF.
Young-old (55–65 years old) and mid-old (66–75 years old) individuals with intact cognitive abilities, particularly those who were initially sedentary, experience benefits on EF from exercise training interventions.

## Introduction

Accumulating evidence suggests that chronic exercise is an important behavior for preventing cognitive decline and impairment in older populations. A positive association between physical activity and cognitive function has been observed in cross-sectional and longitudinal observational studies [1–4]. Further, the positive effects of chronic exercise training interventions on cognitive function have also been described in narrative reviews [5–9], and systematic and meta-analytic reviews of longitudinal studies have revealed that older adults engaged in exercise training interventions are protected against cognitive decline [10, 11].

When considering the specificity of the beneficial effects of exercise on cognition, executive function (EF) is observed to benefit from exercise training interventions as reported in previous meta-analyses [12–14]. In 2003, Colcombe and Kramer [12] conducted a seminal meta-analysis concluding that there was a strong relationship between exercise training and EF. At that time, EF was viewed as a broad construct in the exercise and cognition literature. However, since that time, the operationalization of EF tasks has become more specific and the EF tasks more diversified. EF encompasses basic and underlying cognitive functions for purposeful and goal-directed behavior [15] and EF is related to the neural activity of the prefrontal cortex [16–18]. Chronic exercise has been shown to be associated with activation of the prefrontal cortex [3, 19, 20], and exercise training has been shown to facilitate activation of the prefrontal cortex [21, 22]. EF is not a unitary construct but rather can be divided into (1) core EFs (i.e. inhibition, updating/working memory, switching), and (2) higher-level EFs (i.e. planning/problem solving) [23, 24]. While exercise may benefit these domains differentially, the question has been relatively unexplored in the older population. In addition, although previous narrative and meta-analytic reviews have demonstrated the effect of exercise training on EF broadly, the strength of this evidence is under debate [25, 26]. Importantly, some researchers argue that the evidence for EF benefits from exercise is negligible [25], while others argue that the findings are consistent and positive [26]. Recent meta-analyses support an overall small positive effect of exercise on EF performance by older adults [13, 14] and indicate that the effect is influenced by some aspects of cognition (i.e., sub-domains of memory and EF) [11], but there is no review to date which has explored the extent to which exercise-induced benefits differ across specific sub-domains of EF as currently explored in the exercise and cognition literature.

In addition, previous meta-analyses have also been hampered by experimental and methodological shortcomings. For instance, the American College of Sports Medicine (ACSM) has suggested that principles of exercise prescription aimed at improving health and physical fitness should be guided by the FITT (Frequency, Intensity, Time, Type) principle with specific recommendations made regarding frequency (how many days per week?), intensity (how hard is the exercise?), time (how long does each session last?) and type (what kind of exercise is being performed) [27]; however, previous meta-analyses associated with exercise training interventions and EF have provided relatively little information about these exercise prescription variables.

Last but not least, the effects of exercise training interventions appear to vary according to the characteristics of the sample in question, suggesting that personal demographics might influence the effects of exercise on EF [28–30]. Although two recent meta-analyses reported non-significant differences in effect sizes (ESs) for samples with or without mild cognitive impairment (MCI) [13, 14], other demographic variables (e.g., age, sex, and physical fitness) have not typically been considered as moderators. As such, one way to extend the existing knowledge regarding exercise training interventions and EF in older adults is to conduct a comprehensive meta-analysis in order to broadly examine additional moderators related to the sample characteristics.

Taken together, the present meta-analytic review is primarily aimed at examining the effects of exercise training interventions on EF in older adults who are either cognitively intact or have MCI. In addition, we considered whether these effects were influenced by moderators relative to three main topics: (1) determining whether exercise training interventions have general or selective effects on specific sub-domains of EF; (2) examining if EF is influenced by specific aspects of the prescribed exercise training interventions; and (3) examining if sample characteristics influence the effects of exercise training interventions on EF for older populations.

## Methods

The review’s protocol followed the Preferred Reporting Items for Systematic Review and Meta-Analysis (PRISMA). The meta-analysis was performed following the statement of the PRISMA guidelines [31] and Cochrane Collaboration handbook [32] in order to provide comprehensive and transparent reporting of methods and results.

### Search Strategy

Electronic article searches were conducted for the period between January 2003 and November 2019 in MEDLINE (PubMed) and EMBASE (Scopus) databases. The search terms in this review were a combination of exercise intervention terms (“exercise” OR “physical activity” OR “aerobic” OR “fitness” OR “cardio” OR “VO_2_” OR “Tai Chi” OR “yoga” OR “resistance exercise” OR “weight training”), AND cognitive performance terms (“cognition” OR “cognitive function” OR “cognitive performance” OR “executive function” OR “executive control” OR “inhibition” OR “updating” OR “working memory” OR “switching” OR “planning”), AND aging population terms (“aging” OR “older” OR “55 years old”), AND selected terms regarding experimental designs (“randomized controlled trial” OR “control clinical trial” OR “randomized clinical trial”). We further searched for additional relevant articles in Google Scholar and identified potential studies for inclusion from previous meta-analyses.

### Eligibility Criteria

Studies were included if they met the following criteria: (1) full-length, peer-reviewed study describing a randomized controlled trial (RCT) with human subjects exploring the effects of exercise training interventions on cognition and published in English; (2) participants were men or women aged 55 years and older with normal cognition or diagnosed with MCI; (3) any type of supervised exercise intervention with planned and structured physical activity with the intention of increasing or maintaining physical fitness. Of note, studies that employed one or combined two or more types of exercise modalities as an intervention were included; however, studies involving exercise training interventions combined with other non-exercise activities (e.g. cognitive training, drugs, and video games) and interventions that included unsupervised training sessions were not included; (4) studies that included participants who engaged in no contact, no treatment, waiting list, sham exercise, and alternative active treatments for the comparison condition; and (5) the EF tests had to be administered both at baseline and post-intervention.

### Study Selection and Data Extraction

Two independent reviewers screened the titles and abstracts of all identified articles. Abstracts that matched the inclusion criteria were retrieved as full-text articles. Subsequently, full-text articles were reviewed by the two same reviewers. If there were any disagreements, a third reviewer was consulted until a consensus was achieved.

Two independent reviewers extracted relevant data: study identifiers (e.g. author name, year of publication) and study sample size, EF sub-domains (inhibition, updating/working memory, switching, and planning), exercise prescriptions (frequency, intensity, type, session time, and length), and participants’ characteristics (age, sex, physical fitness level, and cognitive status). If relevant information was not provided in a given study, the first author contacted the authors of the study and made up to three requests for the data by email.

EF domains were generally coded based on four sub-domains: inhibition, updating/working memory, switching, and planning [23, 24] and the specific EF task was also coded (Table 1). EF outcomes were recorded at the baseline and post-intervention time points. For those studies reporting follow-up outcomes, we chose the first time point following the exercise training intervention as the post-intervention time point.

**Table 1:** Classification of executive function assessments

Executive function			
Inhibition	Updating/working memory	Switching	Planning
Stroop Color-Word Stroop-Interference score The California Older Adult Stroop test—interference Auditory Stroop test Hayling sentence completion test Eriksen flanker task—incongruent Go-No-Go Test Stop signal Task The random number generation task	N-back (2 back) Spatial working memory (three item) WCST (number of categories completed) Digit Span task (backward or backward–forward) WAIS-letter number sequencing Verbal fluency-FAS form (number of words) Rey-Osterrieth complex figure test (delay) List sorting task Auditory verbal learning task (verbal fluency) COWAT—verbal fluency Running memory task (total recall) Spatial running span task The list learning delayed recall test	Task switching task (switch, local switch cost, global switch cost) Trail Making Test (part B or part B—part A) Shifting-naming Color trail test Spatial switching Picture switching Arrow switching Attentive Matrices Test D-KEFS verbal fluency (category, switching) The digit-letter task The plus-minus task Dimension-switching task	Tower of London task Six elements test Greenwich test Multiple errands test Hotel test Naturalistic action test RIPA Problem Solving Delay discounting task The Iowa gambling task Cambridge gambling task Raven’s progressive matrices

*WCST* Wisconsin card sorting task, *WAIS* Wechsler adult intelligence scale, *COWAT* Control oral word association test, *D-KEFS* Delis-Kaplan executive function system, *RIPA* Ross information processing assessment

Exercise prescription variables were recorded based upon exercise frequency, intensity, type, session time, and length. Specifically, the exercise frequency was coded as ≤ 2 times/week, 3–4 times/week, or 5–7 times/week as has been done in a previous meta-analysis [14]. The exercise type was coded as aerobic exercise, resistance exercise, Tai Chi and yoga, combined exercise (i.e. the combination of two or more types of training), or other forms of exercise (e.g., dance, coordination exercises). The exercise intensity was coded as moderate (3.00–6.00 METs; 11–13 on the rating of perceived exertion [RPE] of Borg scale; 40–60% HRR/*V*O_2max_; 55–70% HR_max_; 50–70% 1-RM), or vigorous (6.01–9.00 METs; 14–16 on the RPE of Borg scale; 61–85% HRR/*V*O_2max_; 71–90% HR_max_; 71–84% 1-RM) based upon recommendations for exercise intensity terminology [33]. Session time was coded as short (less than 45 min), moderate (45–60 min), or long (over 60 min) [14]. The exercise length was coded as short (1–3 months), medium (4–6 months), or long (over 6 months) [12]. Sample characteristics such as age (young-old/55–65 years; mid-old/66–75 years; old-old/76–85 years) and sex (male; female; both) were coded [12]. We further added variables for physical fitness level (sedentary; fit) and cognitive status [normal; MCI], and the coding of these two variables was determined by descriptions provided in each article.

### Risk of Bias Assessment

Two reviewers independently judged the risk of bias in each study using the Cochrane risk-of-bias tool [32], which was recommended in the existing literature related to exercise and cognition [34, 35] and also conforms to the guidelines of PRISMA [31]. For RCTs, the Cochrane Collaboration Guideline specifies six domains, including sequence generation, allocation concealment, blinding of participants, blinding of assessors, incomplete outcome data, and selective outcome reporting. The potential risk of bias under each domain was evaluated as “low risk”, “high risk”, or “unclear”. Any disagreements were discussed with a third reviewer until a final decision was achieved.

### Statistical Analysis

Version 2.0 of the comprehensive meta-analysis (CMA) software (Englewood, NJ) was utilized for the overall analysis of ES and the subgroup analyses of ES, with statistical significance being defined as a two-sided *p* value of < 0.05.

CMA software was used to compute Hedges’ *g*. The format for this calculation was [(Ex-post) − (Ex-pre)] − [(Con-post) − (Con-pre)]/pooled SD-pre. The ESs were aggregated to calculate an overall ES. A positive ES indicates that the benefits for the exercise group exceed the benefits for the control group and a negative ES represents a deterioration in the performance of the experimental group (that is, those engaged in exercise training interventions) that was larger than the deterioration experienced by the control group or that the control group improved more than the experimental group. According to the criteria of Cohen [36], the interpretation of the magnitude of the effect was such that 0.20–0.49 was interpreted as small, 0.50–0.79 was interpreted as moderate, and more than 0.80 was interpreted as large. Higgins *I*^2^ values were used to assess statistical heterogeneity with possible values ranging from 0 to 100% (1–49% = low, 50–74% = moderate, 75–100% = high heterogeneity). All the ES values were analyzed using fixed-effect models, which were used for calculating pooled ESs (Hedges’ *g*) and 95% confidence intervals (CIs) [37]. Small sample size bias was assessed using Egger’s test and visually examined with a funnel plot of ES relative to standard error.

After calculating an overall ES for EF, subgroup analyses were also performed based on the specific EF domains (inhibition, updating/working memory, switching, planning), exercise prescriptions variables (frequency, intensity, type, session time, and length), and sample characteristics (age, sex, physical fitness level, and cognitive status). Subgroup analyses are not presented when the number of ESs within a level was fewer than ten.

## Results

**Fig. 1:**
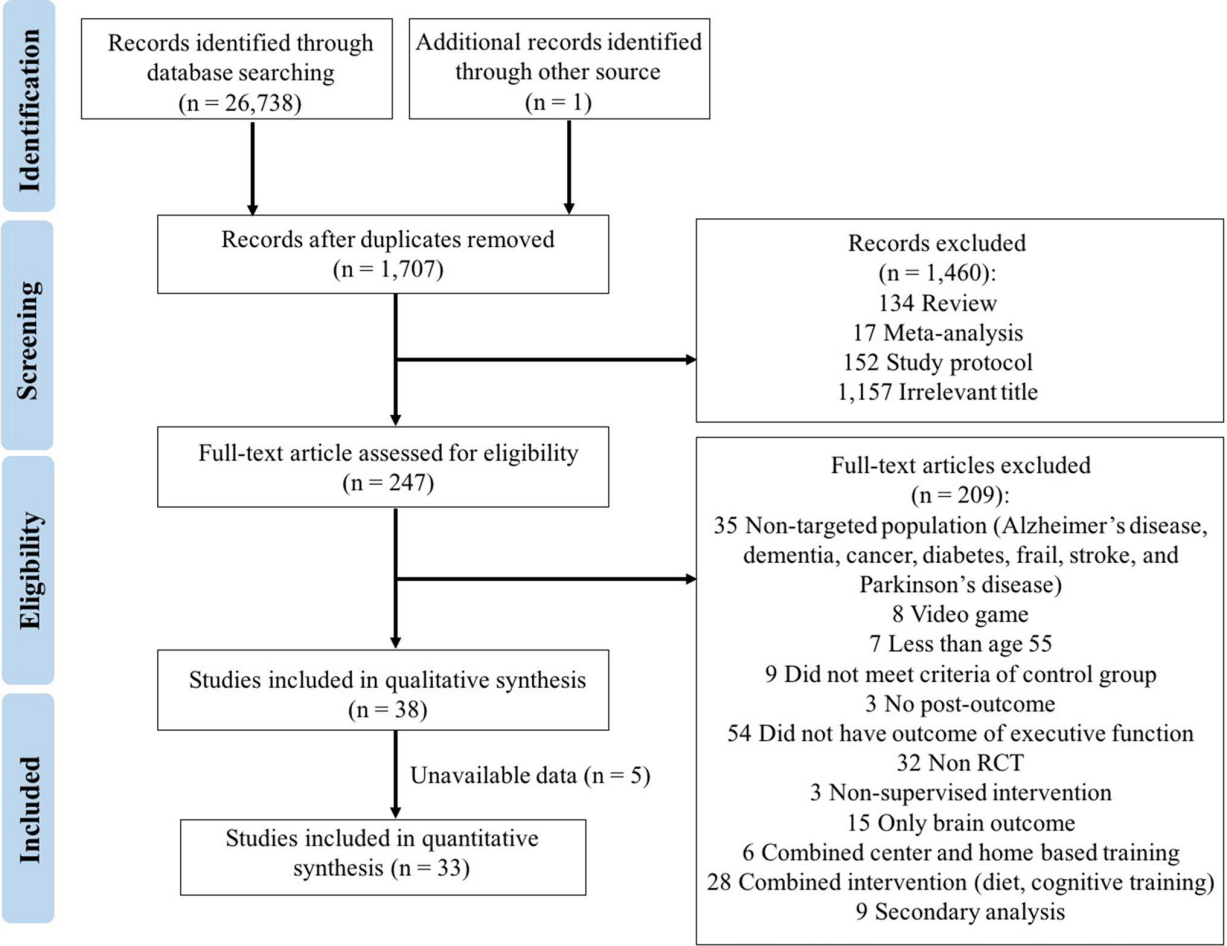
PRISMA flow chart of the study selection process

From an initial search, we identified a total of 26,739 potentially relevant citations, which were further reviewed and led to the retrieval of 247 full-text articles. After evaluating these studies relative to inclusion and exclusion criteria, the meta-analytic review included 38 RCT studies. Of these, the author or authors of six studies with incomplete data were contacted by e-mail. The authors of one study [38] were able to provide additional information, but relevant data were not received for five studies [39–43]. Therefore, 33 articles were included in the quantitative synthesis. The flow of the selection process is summarized in Fig. 1.

### Characteristics of Included Studies

A total of 33 studies were included in this review and the characteristics of each study are presented in Table 2. The sample sizes of the RCTs varied considerably, ranging from 18 to 555 participants. The overall sample size for the review was 7,023 participants including both experimental (*n* = 3606) and control (*n* = 3417) participants.

**Table 2:** Overview characteristics of available evidence from randomized controlled trials on exercise training interventions and executive function

Study	Grouping	EF Task	Exercise characteristics	Sample characteristics
Albinet et al. [64]	Swimming (*n* = 21) Stretching (*n* = 20)	Stroop task RNG Hayling task RST N-back DST Plus-Minus task DLT	Freq.: 2 days/week Int.: 40–65% HRR Type: AE Time: 40 min Length: 21 weeks	Age: 60–75 Sex: Both PF: NR CS: Normal
Albinet et al. [65]	Aerobic (*n* = 12) Stretching (*n* = 12)	WCST	Freq.: 3 days/week Int.: 40–60% HRR Type: AE Time: 40 min Length: 12 weeks	Age: 65–78 Sex: Both PF: Sedentary CS: Normal
Coetsee and Terblanche [66]	Resistance (*n* = 22) Aerobic interval (*n* = 13) Aerobic continuous (*n* = 13) Control (*n* = 19)	Stroop test	Freq.: 3 days/week Int.: 75–100% 10RM (RE) 90–95% HR_max_ (AE—interval) 70–75% HR_max_ (AE—continuous) Type: RE and AE Time: NR (RE) 16 min (90–95% AE—interval) 47 min (70–75% AE—continuous) Length: 16 weeks	Age: 55–75 Sex: Both PF: Fit CS: Normal
Doi et al. [67]	Dance (*n* = 67) Control (*n* = 67)	TMT	Freq.: 1 day/week Int.: NR Type: Dance Time: 60 min Length: 40 weeks	Age: > 70 Sex: Both PF: Fit CS: MCI
Erickson et al. [68]	Aerobic (*n* = 60) Control (*n* = 60)	Spatial memory	Freq.: NR Int.: 60–75% HR_max_ Type: AE Time: 10–40 min Length: 12 months	Age: 65.5–67.6 Sex: Both PF: Sedentary CS: Normal
Ferreira et al. [69]	Walking (*n* = 22) Control (*n* = 22)	WCST	Freq.: 3 days/week Int.: 60–80 HRR Type: AE Time: 40–50 min Length: 6 months	Age: 60–79 Sex: Both PF: Fit CS: Normal
Gothe et al. [70]	Yoga (*n* = 61) Control (*n* = 57)	RST N-back Task switching	Freq.: 3 days/week Int.: NR Type: Yoga Time: 60 min Length: 8 weeks	Age: 62.02 Sex: Both PF: Sedentary CS: Normal
Gothe et al. [71]	Yoga (*n* = 61) Control (*n* = 57)	Task switching running memory span N-back	Freq.: 3 days/week Int.: NR Type: Yoga Time: 60 min Length: 8 weeks	Age: 55–79 Sex: Both PF: Sedentary CS: Normal
Gothe et al. [72]	Yoga (*n* = 61) Control (*n* = 57)	TMT	Freq.: 3 days/week Int.: NR Type: Yoga Time: NR Length: 8 weeks	Age: 62 Sex: Both PF: Sedentary CS: Normal
Iuliano et al. [73]	Resistance (*n* = 20) Cardiovascular (*n* = 20) Postural (*n* = 20) Control (*n* = 20)	RPM Stroop TMT	Freq.: 3 days/week Int.: 80–85% 1RM (RE) 70–80% HRR (AE) Type: RE and AE Time: 30 min Length: 12 weeks	Age: 66.96 Sex: Both PF: Sedentary CS: Normal
Kimura et al. [74]	Strength (*n* = 65) Control (*n* = 85)	Task switching	Freq.: NR Int.: 60% 1RM Type: Combined exercise Time: 1.5 h Length:12 weeks	Age: > 65 Sex: Both PF: Fit CS: Normal
Klusmann et al. [75]	Exercise (*n* = 91) Control (*n* = 76)	VFT Stroop Test TMT	Freq.: NR Int.: NR Type: Combined exercise Time: 90 min Length: 24 weeks	Age: 73.6 Sex: Both PF: Fit CS: Normal
Lam et al. [76]	Physical (*n* = 147) Social (*n* = 131)	LDRT Category Fluency test Digit span TMT	Freq.: 1 day/week Int.: NR Type: Combined exercise Time: NR Length: 48 weeks	Age: > 60 Sex: Both PF: NR CS: MCI
Lazarou et al. [77]	Intervention (*n* = 89) Control (*n* = 65)	VFT ROCFT	Freq.: 2 days/week Int.: NR Type: Dance Time: 60 min Length: 40 weeks	Age: 55–75 Sex: NR PF: Fit CS: MCI
Leckie et al. [78]	Walking (*n* = 47) Control (*n* = 45)	Task switching	Freq.: 3 days/week Int.: 60–75% HRR Type: AE Time: 40 min Length: 48 weeks	Age: 66.82 Sex: Both PF: Sedentary CS: Normal
Liu-Ambrose et al. [79]	RT-1 (*n* = 20) RT-2 (*n* = 15) BAT (*n* = 17)	Flanker task	Freq.: 2 days/week Int.: NR Type: RE Time: NR Length: 48 weeks	Age: 65–75 Sex: F PF: Fit CS: Normal
Liu-Ambrose et al. [80]	RT-1 (*n* = 52) RT-2 (*n* = 54) BAT (*n* = 49)	Stroop test TMT VDS	Freq.: 1–2 days/week Int.: 6–8 RM Type: RE Time: 60 min Length: 48 weeks	Age: 65–75 Sex: F PF: Fit CS: Normal
Lu et al. [81]	Tai Chi (*n* = 15) Control (*n* = 16)	Auditory Stroop test	Freq.: 3 days/week Int.: NR Type: Tai Chi Time: 60 min Length: 16 weeks	Age: > 65 Sex: F PF: Fit CS: Normal
Lu et al. [81]	Dumbbell (*n* = 22) Control (*n* = 23)	TMT Digit Span	Freq.: 3 days/week Int.: NR Type: RE Time: 60 min Length: 12 weeks	Age: > 65 Sex: Both PF: Fit CS: MCI
Mavros et al. [82]	PRT (*n* = 27) Control (*n* = 27)	Category fluency COWAT	Freq.: 2–3 days/week Int.: 80–92% 1RM Type: RE Time: 60–100 min Length: 24 weeks	Age: > 55 Sex: Both PF: Fit CS: MCI
Nagamatsu et al. [83]	Aerobic (*n* = 30) Resistance (*n* = 28) Control (*n* = 28)	SWMT	Freq.: 2 days/week Int.: 6–8 RM (RE) 70–80% HRR (AE) Type: RE and AE Time: 60 min Length: 24 weeks	Age: 70–80 Sex: F PF: Fit CS: MCI
Nguyen and Kruse [84]	Tai Chi (*n* = 48) Control (*n* = 48)	TMT	Freq.: 2 days/week Int.: NR Type: Tai Chi Time: 60 min Length: 24 weeks	Age: 60–79 Sex: Both PF: NR CS: Normal
Nishiguchi et al. [85]	Exercise (*n* = 24) Control (*n* = 24)	TMT	Freq.: NR Int.: NR Type: AE Time: 90 min Length: 12 weeks	Age: > 60 Sex: Both PF: NR CS: Normal
Nocera et al. [86]	Spin (*n* = 10) Control (*n* = 8)	VFT	Freq.: 3 days/week Int.: 50–75% HRR Type: AE Time: 20–45 min Length: 12 weeks	Age: 65–89 Sex: Both PF: Sedentary CS: Normal
Nouchi et al. [87]	Combination (*n* = 32) Control (*n* = 32)	VFT Stroop test Digit Span	Freq.: 3 days/week Int.: 60–80% HR_max_ Type: Combined exercise Time: 30 min Length: 4 weeks	Age: > 60 Sex: Both PF: Sedentary CS: Normal
Oken et al. [51]	Exercise (*n* = 47) Yoga (*n* = 44) Wait list (*n* = 44)	Stroop test	Freq.: 1 day/week and 5 days/week Int.: 70% HR_max_ NR Type: AE Yoga Time: 60 min (AE) 90 min (Yoga) Length: 24 weeks	Age: 65–85 Sex: Both PF: Fit CS: Normal
Prehn et al. [88]	AE (*n* = 11) NAE (*n* = 18)	TMT Digit span Stroop test AVLT	Freq.: 2 days/week Int.: 80%AT Type: AE Time: 45 min Length: 24 weeks	Age: 50–80 Sex: Both PF: Sedentary CS: Normal
Smiley-Oyen et al. [89]	Cardio (*n* = 52) Flexibility (*n* = 53)	GNG Stroop test WCST	Freq.: 3 days/week Int.: 65–80% HRR Type: AE Time: 25–30 min Length: 40 weeks	Age: 65–79 Sex: Both PF: Fit CS: Normal
Suzuki et al. [90]	Exercise (*n* = 25) Control (*n* = 25)	VFT Category fluency Stroop test	Freq.: 2 days/week Int.: 60% HRmax Type: Combined exercise Time: 90 min Length: 48 weeks	Age: 65–93 Sex: Both PF: Sedentary CS: MCI
Sungkarat et al. [91]	Tai Chi (*n* = 33) Control (*n* = 33)	TMT Digit span	Freq.: 3 days/week Int.: NR Type: Tai Chi Time: 50 min Length: 24 weeks	Age: 68.3 (Tai Chi), 67.5 (Control) Sex: Both PF: Sedentary CS: MCI
Tsai et al. [38]	Closed-skill (*n* = 23) Open-skill (*n* = 23) Control (*n* = 23)	N-back Task switching	Freq.: 3 days/week Int.: 70–75% (AE) NR (CE) Type: AE and CE Time: 30 min (AE) 40 min (CE) Length: 24 weeks	Age: 60–80 Sex: M PF: Sedentary CS: Normal
Vaughan et al. [92]	Intervention (*n* = 25) Control (*n* = 23)	TMT LNS Stroop Test COWAT	Freq.: 2 days/week Int.: NR Type: Combined exercise Time: 60 min Length: 16 weeks	Age: 65–75 Sex: F PF: Fit CS: Normal
van Uffelen et al. [93]	Walking (*n* = 77) Placebo activity (*n* = 75)	VFT	Freq.: 2 days/week Int.: 3 MET Type: AE Time: NR Length: 48 weeks	Age: 70–80 Sex: M and F PF: Fit CS: MCI

*NR* not reported, *Freq.* frequency, *Int.* intensity, *PF* physical fitness level, *CS* cognitive status, *M* male, *F* female, *MCI* mild cognitive impairment, *AE* aerobic exercise, *RE* resistance exercise, *CE* coordination exercise, *RNG* the random number generation, *GNG* go and no go, *WCST* Wisconsin card sorting test, *TMT* trail making test, *RST* running span task, *VFT* verbal fluency test, *RPM* Raven’s progressive matrices Test, *DST* the dimension-switching task, *DLT* the digit-letter task, *SWMT* Spatial Working Memory task, *VDS* verbal digit span, *LNS* the letter-number sequencing, *COWAT* control oral word association test, *ROCFT* Rey osterrieth Complex Figure Test, *LDRT* the list learning delayed recall test, *AVLT* auditory verbal learning test, *AT* anaerobic threshold, *MET* metabolic equivalents

In terms of the sub-domains of EF, the largest number of ESs were for measures of updating/working memory (*n* = 42), followed by inhibition (*n* = 27), switching (*n* = 34), and planning (*n* = 4). Regarding exercise prescription variables, the most ESs derived from exercise training interventions offered with a moderate frequency of 3–4 days/week (*n* = 56), performed at a vigorous intensity (*n* = 44), using aerobic exercise (*n* = 45), for a short (≤ 45 min) session time (*n* = 50), and over a medium (4–6 months) length of time (*n* = 49).

The average ages of the participants ranged from 62.0 to 85.9 years, with most of the ESs (*n* = 84) deriving from mid-old adults (66–75 years). The samples in the studies were mostly both male and female participants (*n* = 72), followed by only female (*n* = 21) and only male (*n* = 14). Relatively equal numbers of ESs were calculated when participants were classified as sedentary (*n* = 46) as when they were categorized as physically fit (*n* = 48). Lastly, most ESs derived from individuals with normal cognitive status (*n* = 85), with a smaller number of ESs for participants with MCI (*n* = 22).

**Fig. 2:**
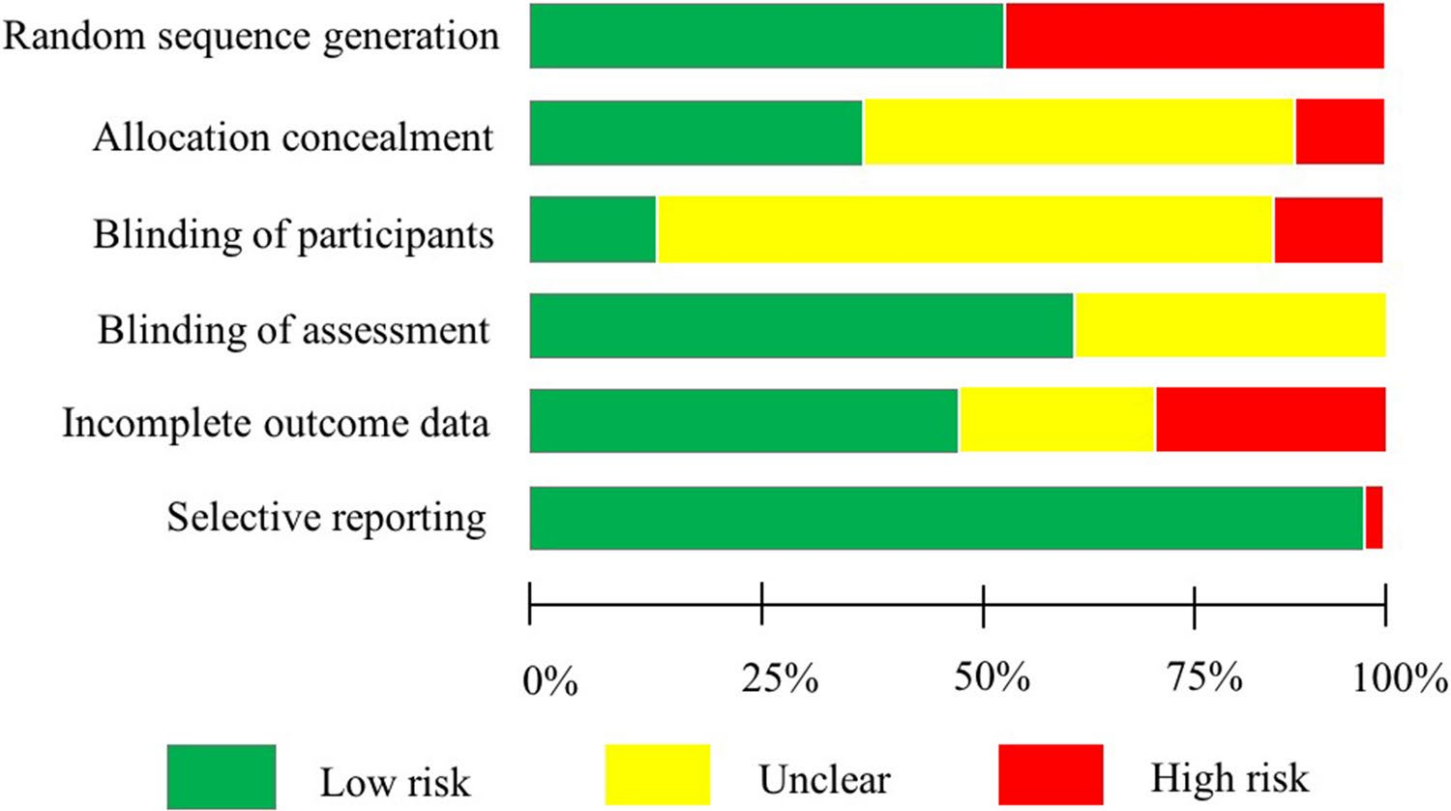
Summary of risk of bias for each item presented as a percentage across all included studies

The summary of the quality assessment data is presented in Fig. 2. The results showed that quality with respect to three of the criteria (e.g. allocation concealment, incomplete outcome data, selective outcome reporting) was low with over 50% of the studies having high or unclear risk.

### Overall Analysis, Heterogeneity and Small Sample Size Bias

**Fig. 3:**
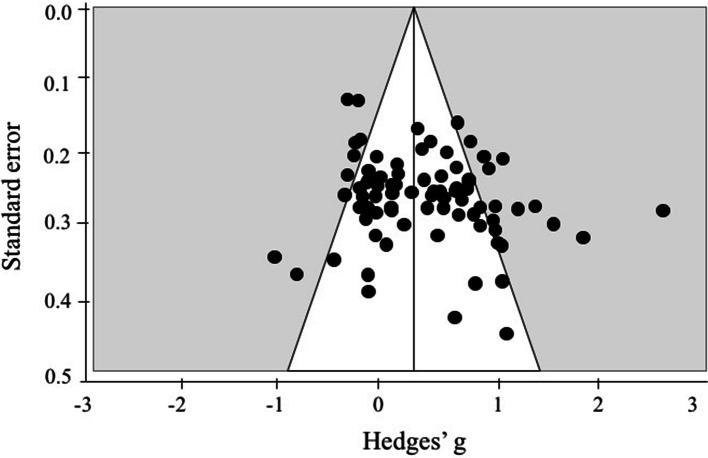
Funnel plot for visual inspection of study bias

The overall ES revealed by the meta-analysis was small but significant and positive (*g* = 0.21, 95% CI 0.17, 0.26, *p* < 0.05) with moderate heterogeneity (*I*^2^ = 59.25) (Table 3). The funnel plot is shown in Fig. 3. Egger’s test and a visual interpretation of the funnel plot suggests that there is no relationship between standard error and ES, suggesting that small sample size has not systematically affected the findings [37].

**Table 3:** Summary of exercise training intervention effect on executive function (EF). Subgroups denote the variables from EF domain, exercise prescription variables and sample characteristics

Overall	*Q*(*df*)	*n* ES	Hedges’ *g* (95% CI)	SE	*I*^2^
	*Q*(106) = 260.09, *p* < 0.01	107	0.21* (0.17, 0.26)	0.02	59.25

	*Q*(*df*)	*n* ES	Hedges’ *g* (95% CI)	SE
EF domains	*Q*(2) = 4.33, *p* > 0.05			
Inhibition		27	0.14* (0.04, 0.24)	0.05
Updating/WM		42	0.19* (0.12, 0.26)	0.04
Shifting		34	0.27* (0.19, 0.36)	0.04
Planning		4	–	–
Exercise prescription variables				
Frequency (week)	*Q*(1) = 10.86, *p* < 0.05			
Low (1–2 times)		46	0.15* (0.08, 0.22)	0.04
Moderate (3–4 times)		56	0.31* (0.24, 0.38)	0.04
Intensity	*Q*(1) = 2.87 *p* > 0.05			
Moderate		25	0.11* (0.00, 0.21)	0.05
Vigorous		44	0.22* (0.14, 0.30)	0.04
Type	*Q*(4) = 26.18, *p* < 0.05			
Aerobic exercise		45	0.14* (0.06, 0.23)	0.04
Resistance exercise		20	0.22* (0.10, 0.33)	0.06
Tai Chi and yoga		14	0.38* (0.27, 0.49)	0.06
Combined exercise		18	0.10* (0.00, 0.19)	0.05
Other forms of exercise		10	0.44* (0.29, 0.60)	0.05
Session time	*Q*(2) = 0.21, *p* > 0.05			
Short (≤ 45 min)		50	0.26* (0.18, 0.33)	0.04
Moderate (45–60 min)		39	0.26* (0.18, 0.33)	0.07
Long (60+ min)		10	0.30* (0.15, 0.44)	0.07
Length	*Q*(2) = 16.64, *p* < 0.05			
Short (1–3 month)		29	0.32* (0.23, 0.41)	0.05
Medium (4–6 month)		49	0.26* (0.18, 0.34)	0.04
Long (> 6 month)		29	0.09* (0.01, 0.17)	0.04
Sample characteristics				
Age	*Q*(2) = 20.64, *p* < 0.05			
Young-old (55–65 yrs)		11	0.30* (0.18, 0.43)	0.06
Mid-old (66–75 yrs)		84	0.25* (0.19, 0.31)	0.03
Old-old (> 76 yrs)		10	− 0.05 (− 0.17, 0.08)	0.06
Sex	*Q*(2) = 5.38, *p* > 0.05			
Only male		14	0.33* (0.18, 0.48)	0.08
Only female		21	0.29* (0.17, 0.40)	0.06
Both		72	0.18* (0.12, 0.23)	0.03
Physical fitness level	*Q*(1) = 10.80, *p* < 0.05			
Sedentary		46	0.33* (0.25, 0.41)	0.04
Fit		48	0.16* (0.09, 0.23)	0.03
Cognitive status	*Q*(1) = 11.44, *p* < 0.05			
Normal		85	0.26* (0.20, 0.32)	0.03
MCI		22	0.08* (0.00, 0.17)	0.05

*n ES* numbers of effect size, *SE* standard error, *I*^*2*^ I square, *yrs* years, *EF* executive function, *WM* working memory, *MCI* mild cognitive impairment

*Represents *p* < 0.05 when comparing the effect size to zero

### Subgroup Analysis

#### EF Sub-Domains

The results of the subgroup analyses are shown in Table 3. Four EF sub-domains were included in the meta-analytic review; however, the number of ESs for the sub-domain of planning was fewer than 10, which did not match the criteria for further analysis. For other EF sub-domains, the results indicated no significant differences among inhibition, updating/working memory, and shifting [*Q*(2) = 4.33, *p* > 0.05].

#### Exercise Prescription Variables

The frequency of exercise training interventions significantly moderated the effect of exercise on EF [*Q*(1) = 10.86, *p* < 0.05]. The results of the subgroup analysis indicated that the ES for older adults engaged in exercise training interventions of moderate frequency (3–4 times per week) (*g* = 0.31, *p* < 0.05) was larger than that for low frequency (1–2 times per week) (*g* = 0.15, *p* < 0.05). The subgroup analysis indicated that type of exercise training [*Q*(4) = 26.18, *p* < 0.05] significantly moderated the effect of exercise on EF, showing ESs for older adults engaged in exercise training interventions of other forms (*g* = 0.44, *p* < 0.05) were largest, followed by Tai Chi and yoga (*g* = 0.38, *p* < 0.05), resistance exercise (*g* = 0.22, *p* < 0.05), aerobic exercise (*g* = 0.14, *p* < 0.05), and combined exercise (*g* = 0.10, *p* < 0.05). In addition, the results of subgroup analysis also showed that exercise length [*Q*(2) = 16.64, *p* < 0.05] significantly moderated the effect of exercise on EF, revealing ESs were largest for short length (1–3 month) exercise training (*g* = 0.32), followed by mid length (4–6 month) exercise training (*g* = 0.26), and long length (> 6 month) exercise training (g = 0.09).

There were no significant differences among the ESs based upon intensity level [*Q*(1) = 2.87, *p* > 0.05] and exercise session time [*Q*(2) = 0.21, *p* > 0.05].

#### Sample Characteristics

The subgroup analysis indicated age significantly moderated the effects of exercise on EF [*Q*(2) = 20.64, *p* < 0.05], such that ESs for young-old participants (55–65 years old) (*g* = 0.30, *p* < 0.05) and mid-old participants (66–75 years old) (*g* = 0.25, *p* < 0.05) were significant. In contrast, studies with old-old participants (more than 76 years old) exhibited negligible ESs (*g* = − 0.05, *p* > 0.05). Additionally, the subgroup analysis indicated significant differences in the ESs as a function of physical fitness levels [*Q*(1) = 10.80, *p* < 0.05] and indicated that sedentary participants (*g* = 0.33, *p* < 0.05) had a greater relative improvement in EF than fit participants (*g* = 0.16, *p* < 0.05). Furthermore, the subgroup analysis also showed a significant difference in the ESs as a function of cognitive status [*Q*(1) = 11.44, *p* < 0.05], suggesting that ESs were larger for participants with cognitively normal (*g* = 0.26, *p* < 0.05) as compared to those with MCI (*g* = 0.08, *p* < 0.05).

The results of the subgroup analysis showed no significant differences as a function of sex [*Q*(2) = 5.38, *p* > 0.05].

## Discussion

To our knowledge, this is the first meta-analysis of RCTs to have investigated the effects of exercise training interventions specifically on EF and its sub-domains among older adults. This is important because of the evidence that the largest benefits of exercise training interventions for older adults may be apparent for EF [12]. In addition, no previous meta-analytic reviews have examined if exercise training influences specific domains of EF [13, 14], and it is critical to advance our understanding of moderators important for predicting the magnitude of these benefits.

In this meta-analysis, we analyzed the results of 33 RCT studies and demonstrated that exercise training interventions improve EF with a positive, significant, small ES. According to the overall results of the current meta-analytic review, we suggest that exercise training interventions are an effective approach to improving EF in older populations, a view which is generally consistent with the results of several previous meta-analyses [12, 14, 44]. Although the results of this review were similar to those of previous meta-analyses in terms of the pattern of positive effects of exercise training interventions on EF, it is important to recognize that the overall effect obtained was considered small in magnitude. This is a contrast to the moderate ES reported in Colcombe and Kramer [12] and is more consistent with the small ES shown in two other meta-analyses [14, 44].

### EF Sub-Domains

Despite previous studies showing the positive effects of exercise training interventions on EF in older adults [12, 14, 44], this meta-analysis provides an important extension to the literature by exploring the effects of exercise training interventions on sub-domains of EF. The results of the subgroup analysis indicated that older adults engaged in exercise training interventions exhibited small to moderate significant improvements within three specific domains of EF. These domains were inhibition, updating/working memory, and switching. Consistent with a recent meta-analytic review that examined the effects of regular exercise training on EF in children and reported small improvements in inhibition [45], we showed small effects of exercise training interventions on inhibition in older adults. In addition, we also demonstrated that exercise training interventions for older adults resulted in positive effects on other aspects of EF (i.e., updating/working memory, switching). The findings overall suggest that exercise training interventions influenced multiple aspects of EF.

However, we were not able to present a robust average ES for planning due to the small number of ESs available (*n* = 4), which did not match the criteria for inclusion in this meta-analytic review. Despite the small number of previous studies that have focused on planning, we suggest that the planning aspect of EF should be studied in the future due to the domain being equally important as other EFs (e.g. inhibition, updating/working memory, switching) for older populations [46]. Additionally, several cross-sectional and intervention studies have shown a positive relationship between exercise training interventions and planning in children [47–50], suggesting the possibility that exercise training interventions may also benefit from planning in an older population.

### Exercise Prescription Variable

The present meta-analytic review constitutes an important step in evaluating the effects of another group of moderators (e.g. exercise prescription) on the effects of exercise training interventions on EF. The findings indicated that program frequency is a moderator that influences EF effects, such that larger benefits are evident with moderate frequency (3–4 times) exercise as compared to low frequency (1–2 times) exercise. Unfortunately, there were an insufficient number of studies (*n* = 1) [51] testing the effects of high frequency exercise (more than 5 times per week) to allow for a statistical examination of the ES for these studies. Although we found differences in ESs as a function of the frequency of the exercise sessions, it is important to acknowledge that our finding of a positive benefit for both low and moderate frequency exercise is consistent with the results of Northey et al. [14] who suggested that exercise performed at low and moderate frequencies benefits global cognition.

Another issue addressed in this review was examining the effect of exercise intensity on the EF outcomes. The results of this meta-analysis showed a small ES of exercise training intervention on EF regardless of intensity levels, suggesting that exercise training interventions of moderate or vigorous intensity yielded positive improvements in terms of EF. Consistent with a previous meta-analysis which demonstrated that moderate and vigorous intensity exercise training interventions might be an effective alternative for improving global cognitive function [14], our findings further expand the understanding of the positive effect of such interventions specifically on EF among older adults.

The current meta-analysis indicated that exercise training interventions yielded positive improvements in EF regardless of the exercise type, thereby confirming the results of a previous meta-analysis of RCTs in older adults [12]. Importantly, however, our results showed that other forms of exercise training (e.g., dance, coordination exercises) exhibited the largest ES, followed by Tai Chi and yoga, resistance exercise, combined exercise, and aerobic exercise. Notably, these results should be interpreted with caution due to the relatively small number of ESs for all exercise types (i.e., Tai Chi and yoga, combined exercise, resistance, and other) other than aerobic exercise.

For aerobic exercise, our results were similar to Angevaren et al. [52] who reported that RCTs of aerobic exercise training were associated with positive improvements in cognition (e.g. attentional processes, cognitive speed, and motor functions). Similarly, aerobic exercise in this review was found to produce small improvements in EF among older populations.

We also observed positive improvements in EF when older adults engaged in resistance exercise training. These results are consistent with a previous meta-analytic review suggesting that resistance exercise yields positive improvements in terms of the cognitive functions of older adults [14]. Furthermore, we included studies testing the effects of Tai Chi and yoga, since we considered these exercises to have the same functions in terms of enhancing motor fitness (e.g. balance, flexibility, and agility). Importantly, the cognitive benefits of these exercise programs were significantly larger than were observed for aerobic exercise. These types of exercise might be considered to be unique in that they are sometimes categorized as mind–body exercises and have been shown to influence different aspects of physical fitness (e.g., flexibility, balance) as compared to aerobic exercise [35]. Although a previous meta-analysis revealed no effects of yoga on global cognitive function [14], our meta-analysis provides evidence of higher ES for EF from the prescription of both Tai Chi and yoga in older populations. This is an important finding with regard to exercise training interventions, because Tai Chi and yoga have been described as being more suitable than some other types of exercise for older adults because of the emphasis on slow controlled movements that result in a minimal risk of injury [53, 54].

A previous meta-analysis showed that combined exercise programs have moderate effects on global cognitive function [12]; the results of this review indicated that combined exercise programs have a small effect on EF. Combined exercise in the present review was defined as the combination of two or more types of exercise training as an intervention; therefore, it was more broad than the categories used in previous meta-analyses which only combined aerobic exercise and resistance exercise [12, 14]. Furthermore, we also included additional exercise types (e.g. dance, coordination exercise) in the review (categorized as ‘other’) and the ESs were the largest observed. Given that it is important to provide exercise guidelines for older populations, including strategies to maintain and improve EF, future RCTs of exercise training interventions focusing on combined exercise trainings are needed to confirm the findings of the present review.

Lastly, this meta-analytic review also examined differences in ES as a function of the time of exercise training interventions. The results of the subgroup analyses significant improvement in EF as a function of the session time or length of the program across studies with session times that ranged from ≤ 45 to > 60 min and program lengths from 1 months to more than 6 months. This suggests that within these ranges, exercise training interventions of any session time and length result in positive effects for EF in older adults, as suggested in previous reviews [12, 14]. Although these meta-analytic results suggest that the specifics in terms of the time of exercise training interventions are not critical for demonstrating improvements in EF, it is important for dose–response studies to be conducted to more formally test the effects of exercise time on the EF benefits observed in an older population.

### Sample Characteristics

A previous meta-analysis reported that RCTs of aerobic exercise training resulted in moderate to large positive effects on global cognition among all ages of older adults [12]. The results of the present meta-analysis are also positive but show smaller effects on EF. Given that significant differences were observed between age groups, these findings highlight that exercise training interventions are a strategy to benefit EF in specific age groups of older adults. This might then suggest that exercise is particularly beneficial for EF in relatively younger groups of older adults. However, these results should be interpreted with caution because the number of ESs for the mid-old (66–75) participants was considerably larger than the young-old (55–65) and old-old (> 76) participants.

Previous studies have shown that some psychological factors that are associated with biological sex (e.g., steroid hormones, brain-derived neurotrophic factor) may impact cognitive performance [55–57] Thus, we compared studies using single sex samples (male or female) with studies including both sexes. Our findings showed positive effects of exercise on EF in male and female participants tested in single-sex study samples and positive improvements in EF in studies combining both male and female participants. These findings were consistent with those of a previous meta-analysis [12], which suggested no different benefits in cognitive function in those studies including single sex (i.e. male and female) or both sexes. However, this is in contrast to a recent meta-analysis which suggested that some types of exercise training (aerobic exercise, resistance exercise, and combined exercises) had positive and large ESs in studies including higher percentages of female participants [13, 58]. An important feature differentiating this review from previous reviews is that our data focus only on EF and suggest that exercise training interventions can benefit EF in both male and female participants, with the ES being similar for both sexes.

Another issue was to specifically examine the effects of exercise training interventions on EF in older adults with different physical fitness levels. Interestingly, the subgroup analysis suggested that the ES for sedentary participants was significantly greater than that for participants with higher physical fitness levels. Accordingly, this would imply that older adults with a sedentary lifestyle who participate in regular exercise training would experience greater benefits in terms of EF. This finding supports previous reviews which suggested that exercise in those with lower physical fitness levels might be more beneficial to EF as compared to such activity in those with high physical fitness levels [59, 60]. However, while the ESs for sedentary and fit participants were significantly different, the findings of this review suggested a positive effect on EF regardless of fitness levels.

Regarding cognitive status, our result is consistent with previous findings, showing significant improvement in EF among cognitively normal older adults and among older adults with MCI [14, 61–63]. Although the ES of older adults with normal cognition is significant higher than those with MCI, the implication of this finding is that exercise can be beneficial even for older adults who have begun to show evidence of clinical cognitive impairment. Taken together, previous and present meta-analyses suggest that exercise training interventions have the capacity to improve EF in older adults with MCI.

### Strengths and Limitations

In this meta-analytic review, the primary strength was the inclusion of RCT studies. Because the inclusion criteria required an experimental design, the results may be interpreted in terms of causal relationships. Another strength is the consideration of various key moderators such as the specific EF sub-domain, exercise prescription variables, and sample characteristics. This provides a much clearer picture of the complexity of the relationship between exercise training interventions and EF than presented in previous reviews.

The present meta-analysis had four limitations, which should be considered in future research. First, because our goal was to update our knowledge of the effects of exercise on cognitive performance looking specifically at sub-domains of EF, we limited our search to publications since 2003 when Colcombe and Kramer first identified an overall positive ES for studies focused on older adults and EF [12]. Second, our data indicated that the quality of the studies reviewed was generally poor. Although all of the studies were RCTs, in general the studies did not control for potential risks of bias or failed to report the extent to which they did control for these risks and, hence, are open to alternative interpretations of the findings. Third, some moderators (i.e., EF sub-domain: planning; exercise frequency: high) were excluded from the analysis because fewer than 10 ESs were available overall and there were some moderator levels that were exactly at this minimum number of ESs (e.g., length of exercise session > 60 min). Although a meta-analysis does not require a specific number of ESs, a small number of ESs may limit the precision of pooled estimates and the power to detect effects, thus necessitating a priori decisions of a minimum number of ES for inclusion in analyses. Setting a limit of 10 ESs provides some control over this concern, but findings for moderator levels with smaller number of ESs must be considered to be somewhat tentative. Finally, previous reviews examining the outcome of both cognitive and physical function have suggested that the focus of a study in terms of its primary and secondary outcomes may moderate the results of the effects of exercise on cognition [1]. However, most of the studies included in this meta-analytic review examined EF as a primary outcome, making it impossible to examine primary versus secondary outcome as a moderator. In addition, because studies are often powered to detect only the primary outcomes, it may be valuable in the future to consider this as a potential moderator.

## Conclusion

The results of this review demonstrated that exercise training interventions are a promising way to promote overall EF, including inhibition, updating/working memory, and shifting, with small positive effects. Additionally, we observed that exercise training interventions of low and moderate frequency yielded improvements in EF, while also finding positive effects for all types of exercise training and positive effects for interventions at any intensity (i.e., moderate, vigorous) and length (i.e., 1 months to more than 6 months). Regardless of the exercise session time, the size of the effects of exercise training interventions on EF were similar. Last, the results suggested that, regardless of health status, older individuals who were initially sedentary appeared to benefit from exercise training interventions in terms of EF.

## Data Availability

All data generated or analyzed during this review are included in this published article (and its supplementary information files).
